# Obituary

**Published:** 1914-10

**Authors:** 


In the September issue, we published an obituary of Dr. Eugene A. Smith. As Dr. Smith had been promiment in military affairs, we append the following schedule of his service, compiled from the official records by Lt. Col. Albert H. Briggs, M. D., of Buffalo.

Eugene Alfred Smith: Private, Company F, 65th Regt., N. G. N. Y., October 15th, 1881. Promoted to Corporal, December 20th, 1882; promoted to Sergeant, May 1st, 1885; and made First Sergeant, January 9th, 1888; and commissioned Second Lieutenant, January 23d, 1888. November 11th, 1889, promoted to the rank of First Lieutenant and transferred to Company B, 65th Regt. On March 31st, 1890 he was commissioned Captain of the same company. He served as the commanding officer of Co. B until December 13th, 1895, when he was transferred to the Regimental Staff as Inspector of Rifle Practice, with original rank. May 1st, 1899 the Regiment was transferred from the National Guard Service of New York, to the United States Service, as a volunteer Regiment to serve for two years, or during the war with Spain, and Captain Smith was promoted to the rank of Major U. S. Vol., May 17th, 1898 and served until November 19th, 1898, when the Regiment was mustered out of the United States Service at the close of the war, and returned to the National Guard Service.

July 12, 1899, Major Smith resigned. February 10, 1904, he was appointed Surgeon of the Fourth Brigade with the grade of Lieutenant-Colonel. He served in this grade, until the re-organization of the medical corps, to comply with the rules of the War Department, when he was rendered supernumerary, January 21st, 1908, and on the same date was re-commissioned Major of the Medical Corps and assigned to duty as Chief Medical Officer of the Fourth Brigade, N. G. N. Y. He served in this capacity until his failing health compelled his retirement. He was placed on the retired list, January 28, 1910.

